# Supplementary material for: Methyl Canthin-6-one-2-carboxylate Inhibits the Activation of the NLRP3 Inflammasome in Synovial Macrophages by Upregulating Nrf2 Expression
Source: Curr Issues Mol Biol. 2025 Jan 9;47(1):38. doi: 10.3390/cimb47010038 (PMC11763762; doi:10.3390/cimb47010038)
Supplement: Supplementary file 1 [file cimb-47-00038-s001.zip › Cant MS unedited blots.pdf]

Figure 2B

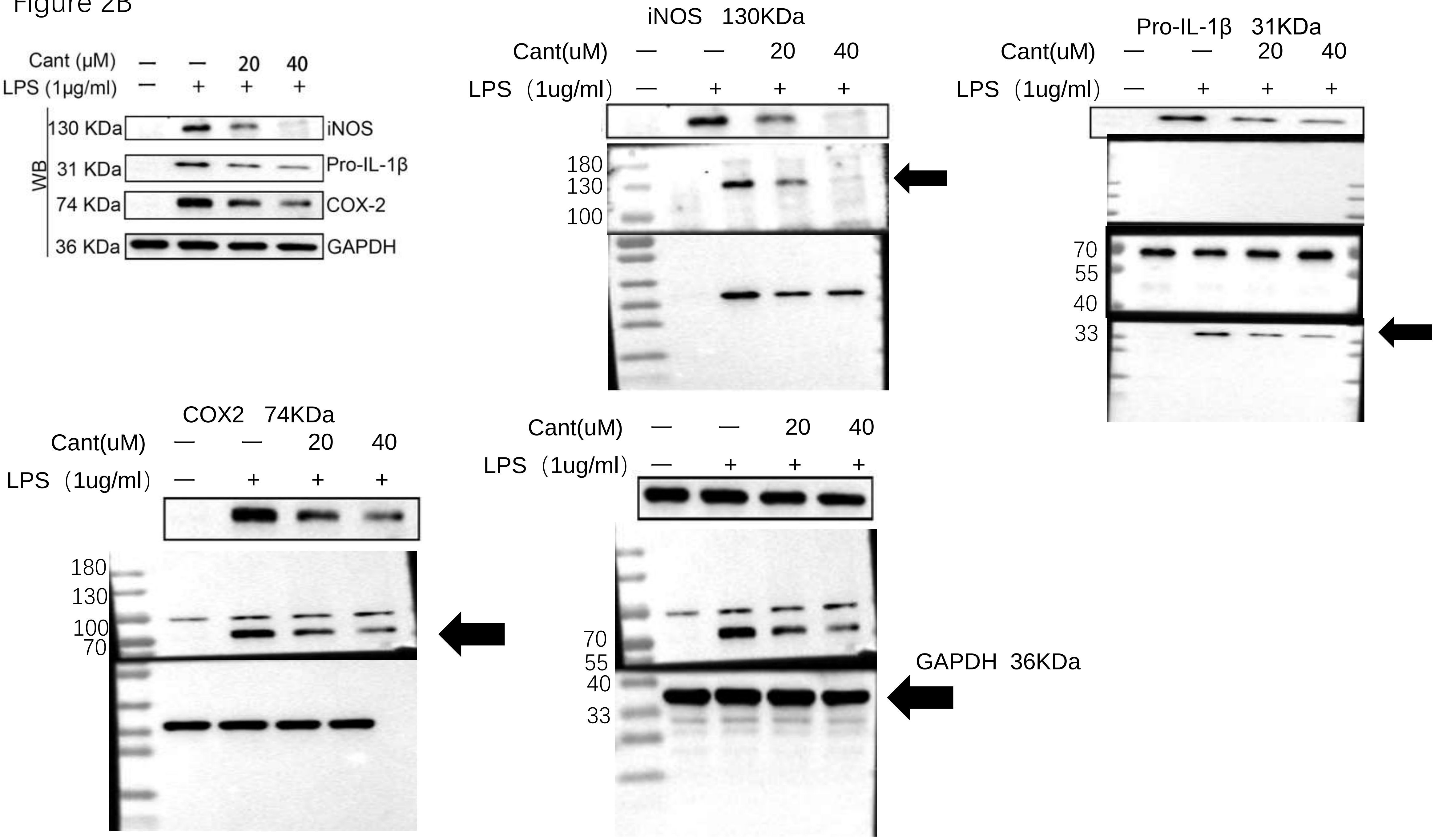

Cant (μM) — — 20 40
LPS (1μg/ml) — + + +
WB
130 KDa iNOS
31 KDa Pro-IL-1β
74 KDa COX-2
36 KDa GAPDH
iNOS 130KDa
Cant(uM) — — 20 40
LPS（1ug/ml） — + + +
180
130
100
Pro-IL-1β 31KDa
Cant(uM) — — 20 40
LPS（1ug/ml） — + + +
70
55
40
33
COX2 74KDa
Cant(uM) — — 20 40
LPS（1ug/ml） — + + +
180
130
100
70
Cant(uM) — — 20 40
LPS（1ug/ml） — + + +
70
55
40
33
GAPDH 36KDa

Figure 2E

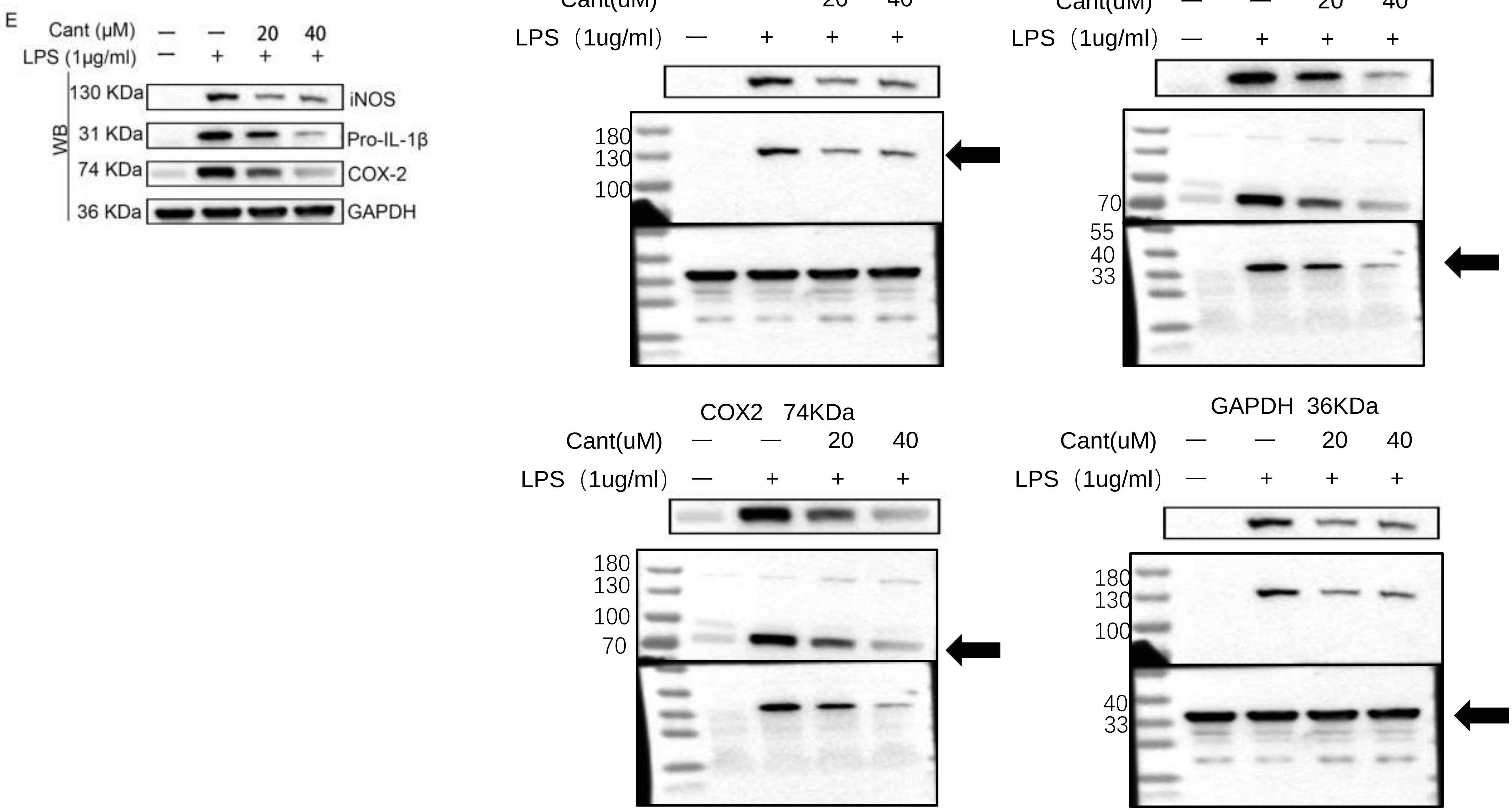

E
Cant (μM) — — 20 40
LPS (1μg/ml) — + + +
WB
130 KDa iNOS
31 KDa Pro-IL-1β
74 KDa COX-2
36 KDa GAPDH
iNOS 130KDa
Cant(uM) — — 20 40
LPS（1ug/ml） — + + +
180
130
100
Pro-IL-1β 31KDa
Cant(uM) — — 20 40
LPS（1ug/ml） — + + +
70
55
40
33
COX2 74KDa
Cant(uM) — — 20 40
LPS（1ug/ml） — + + +
180
130
100
70
GAPDH 36KDa
Cant(uM) — — 20 40
LPS（1ug/ml） — + + +
180
130
100
40
33

Figure 3A

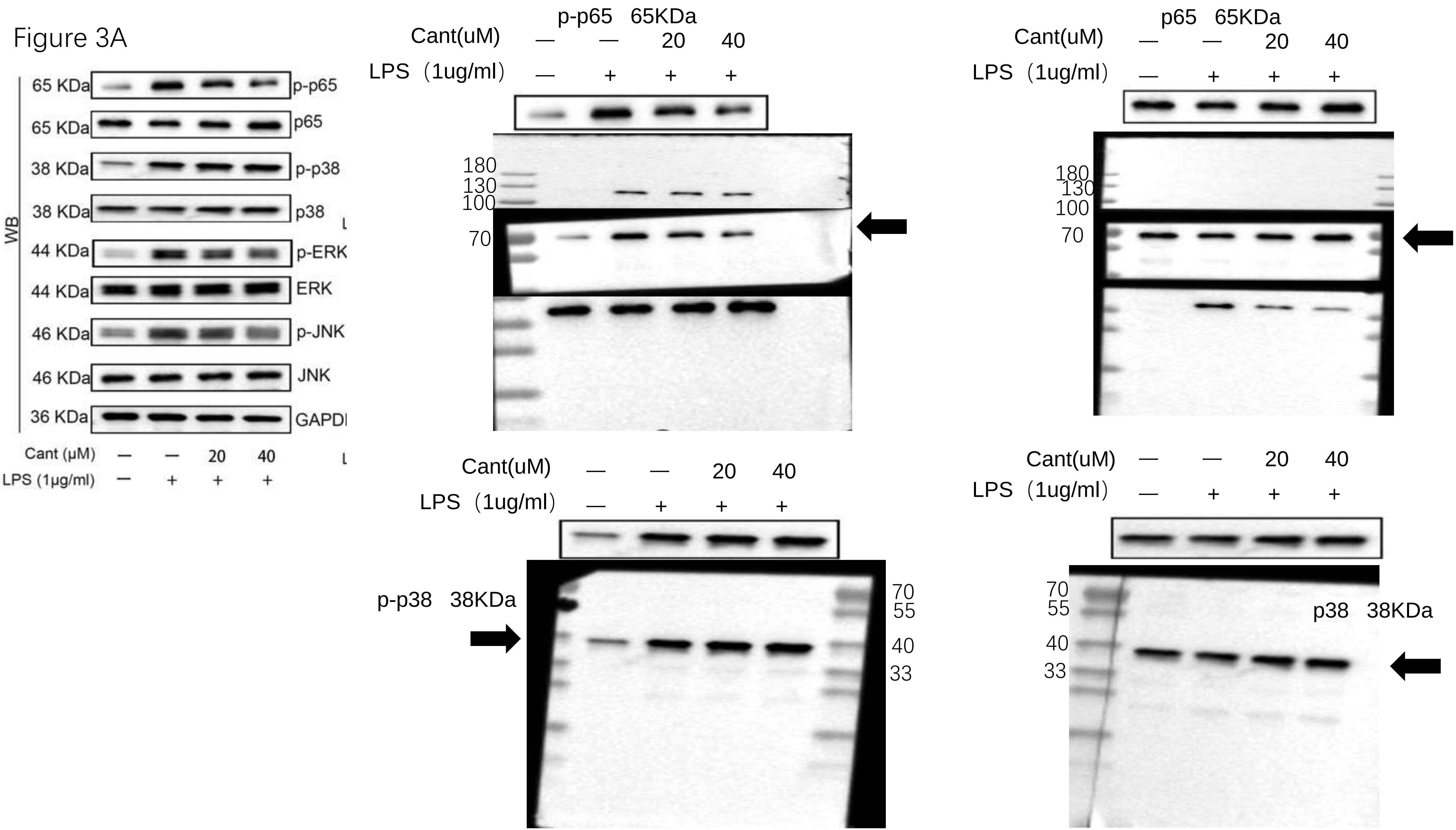

65 KDa
p-p65
65 KDa
p65
38 KDa
p-p38
38 KDa
p38
44 KDa
p-ERK
44 KDa
ERK
46 KDa
p-JNK
46 KDa
JNK
36 KDa
GAPD
WB
Cant (μM) — — 20 40
LPS (1μg/ml) — + + +
p-p65 65KDa
Cant(uM) — — 20 40
LPS（1ug/ml） — + + +
180
130
100
70
p65 65KDa
Cant(uM) — — 20 40
LPS（1ug/ml） — + + +
180
130
100
70
Cant(uM) — — 20 40
LPS（1ug/ml） — + + +
p-p38 38KDa
70
55
40
33
Cant(uM) — — 20 40
LPS（1ug/ml） — + + +
70
55
40
33
p38 38KDa

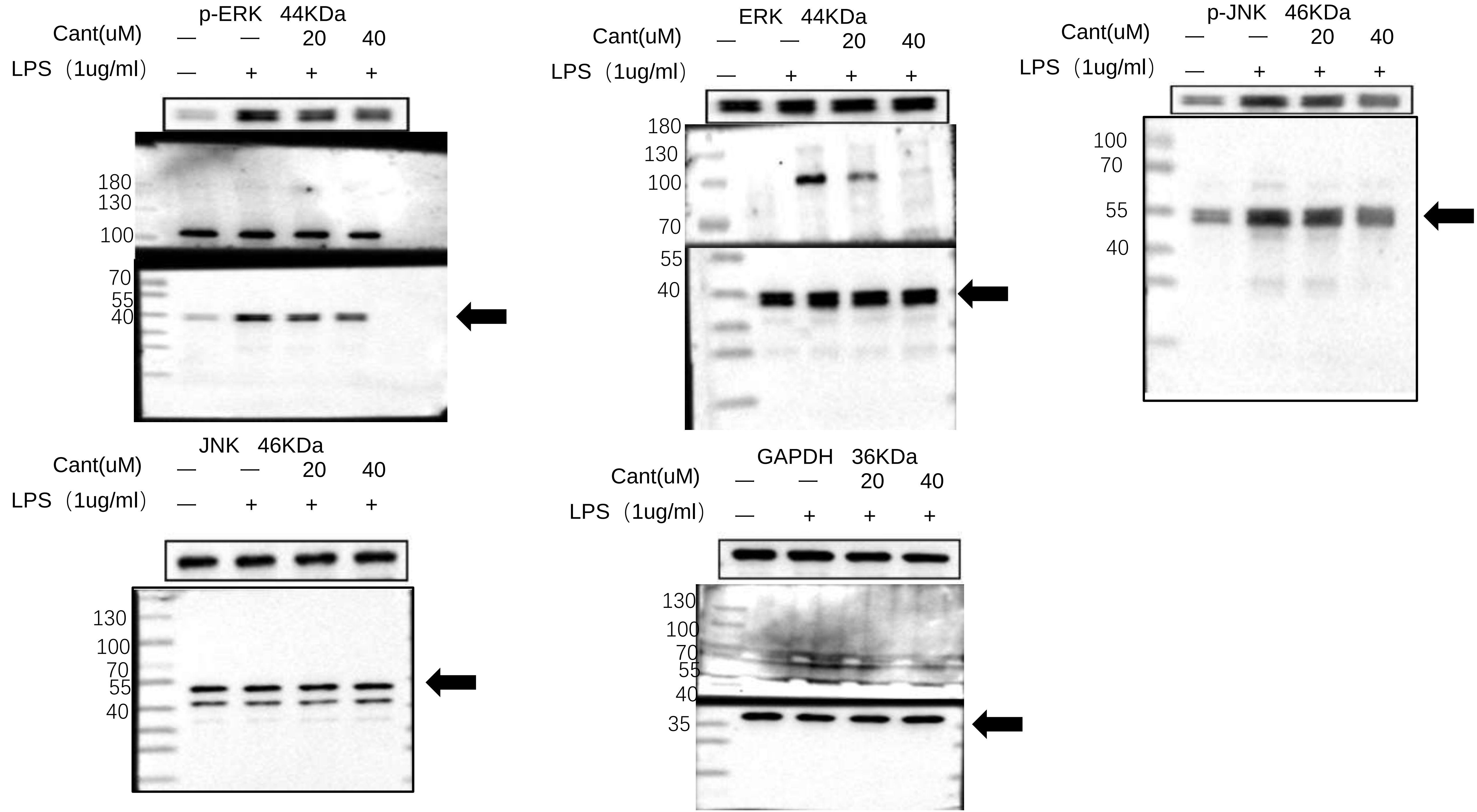
p-ERK 44KDa
Cant(uM) — — 20 40
LPS（1ug/ml） — + + +
180
130
100
70
55
40
ERK 44KDa
Cant(uM) — — 20 40
LPS（1ug/ml） — + + +
180
130
100
70
55
40
p-JNK 46KDa
Cant(uM) — — 20 40
LPS（1ug/ml） — + + +
100
70
55
40
JNK 46KDa
Cant(uM) — — 20 40
LPS（1ug/ml） — + + +
130
100
70
55
40
GAPDH 36KDa
Cant(uM) — — 20 40
LPS（1ug/ml） — + + +
130
100
70
55
40
35

Figure 3C

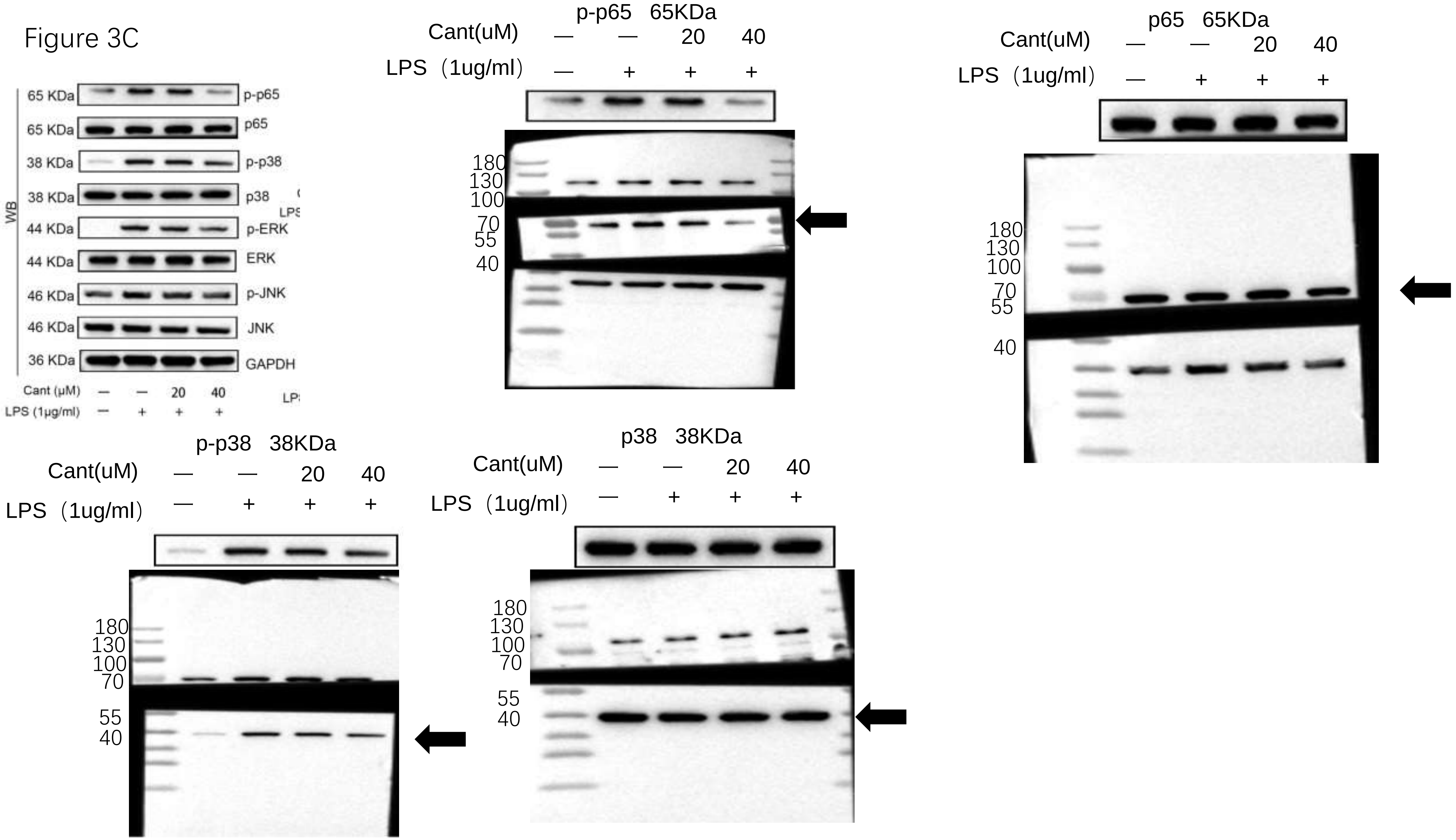

65 KDa p-p65
65 KDa p65
38 KDa p-p38
38 KDa p38
44 KDa p-ERK
44 KDa ERK
46 KDa p-JNK
46 KDa JNK
36 KDa GAPDH
WB
Cant (μM) — — 20 40
LPS (1μg/ml) — + + +
p-p65 65KDa
Cant(uM) — — 20 40
LPS（1ug/ml） — + + +
180
130
100
70
55
40
p65 65KDa
Cant(uM) — — 20 40
LPS（1ug/ml） — + + +
180
130
100
70
55
40
p-p38 38KDa
Cant(uM) — — 20 40
LPS（1ug/ml） — + + +
180
130
100
70
55
40
p38 38KDa
Cant(uM) — — 20 40
LPS（1ug/ml） — + + +
180
130
100
70
55
40

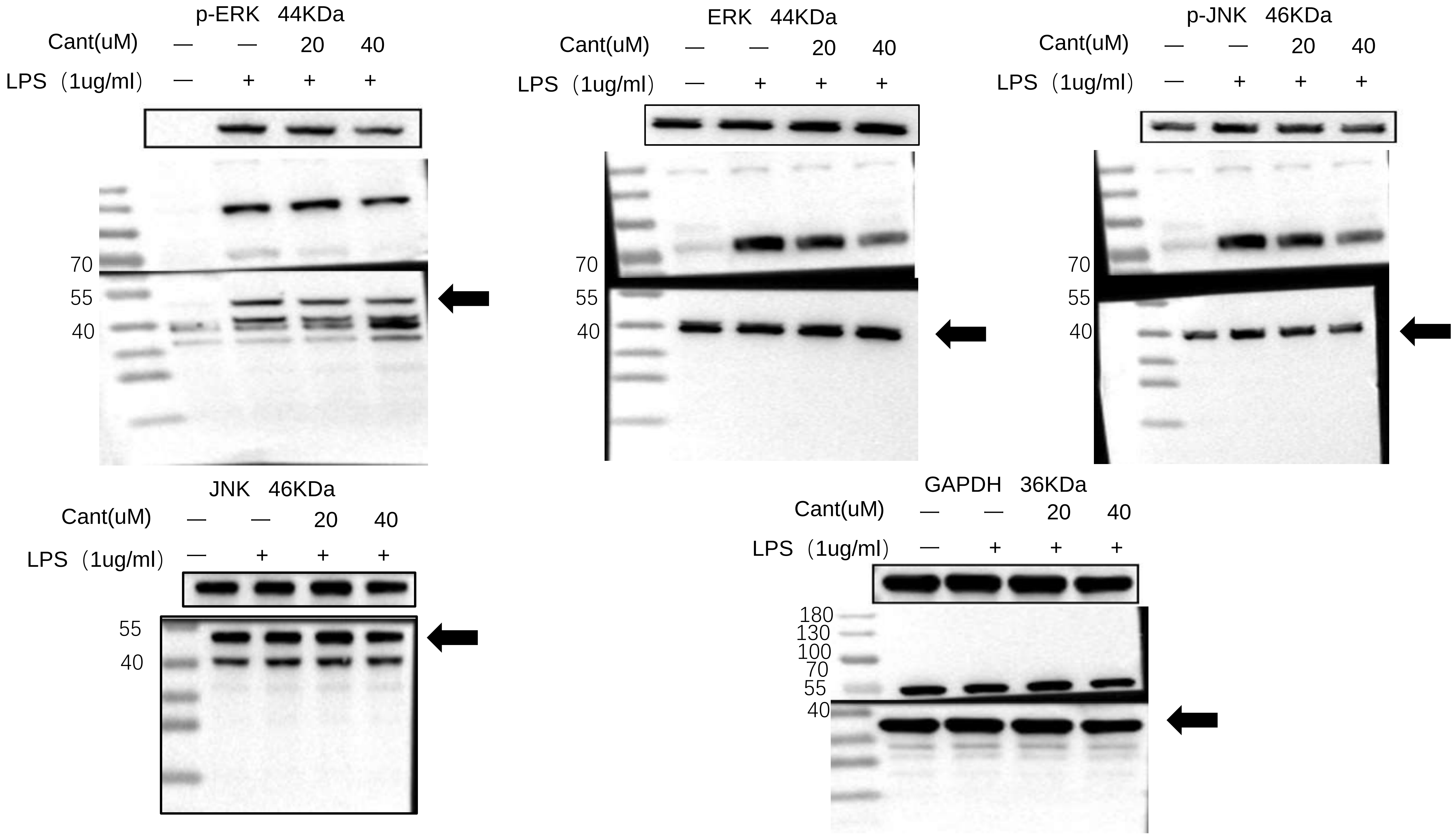

p-ERK 44KDa
Cant(uM) — — 20 40
LPS（1ug/ml） — + + +
70
55
40
ERK 44KDa
Cant(uM) — — 20 40
LPS（1ug/ml） — + + +
70
55
40
p-JNK 46KDa
Cant(uM) — — 20 40
LPS（1ug/ml） — + + +
70
55
40
JNK 46KDa
Cant(uM) — — 20 40
LPS（1ug/ml） — + + +
55
40
GAPDH 36KDa
Cant(uM) — — 20 40
LPS（1ug/ml） — + + +
180
130
100
70
55
40

Figure 3E

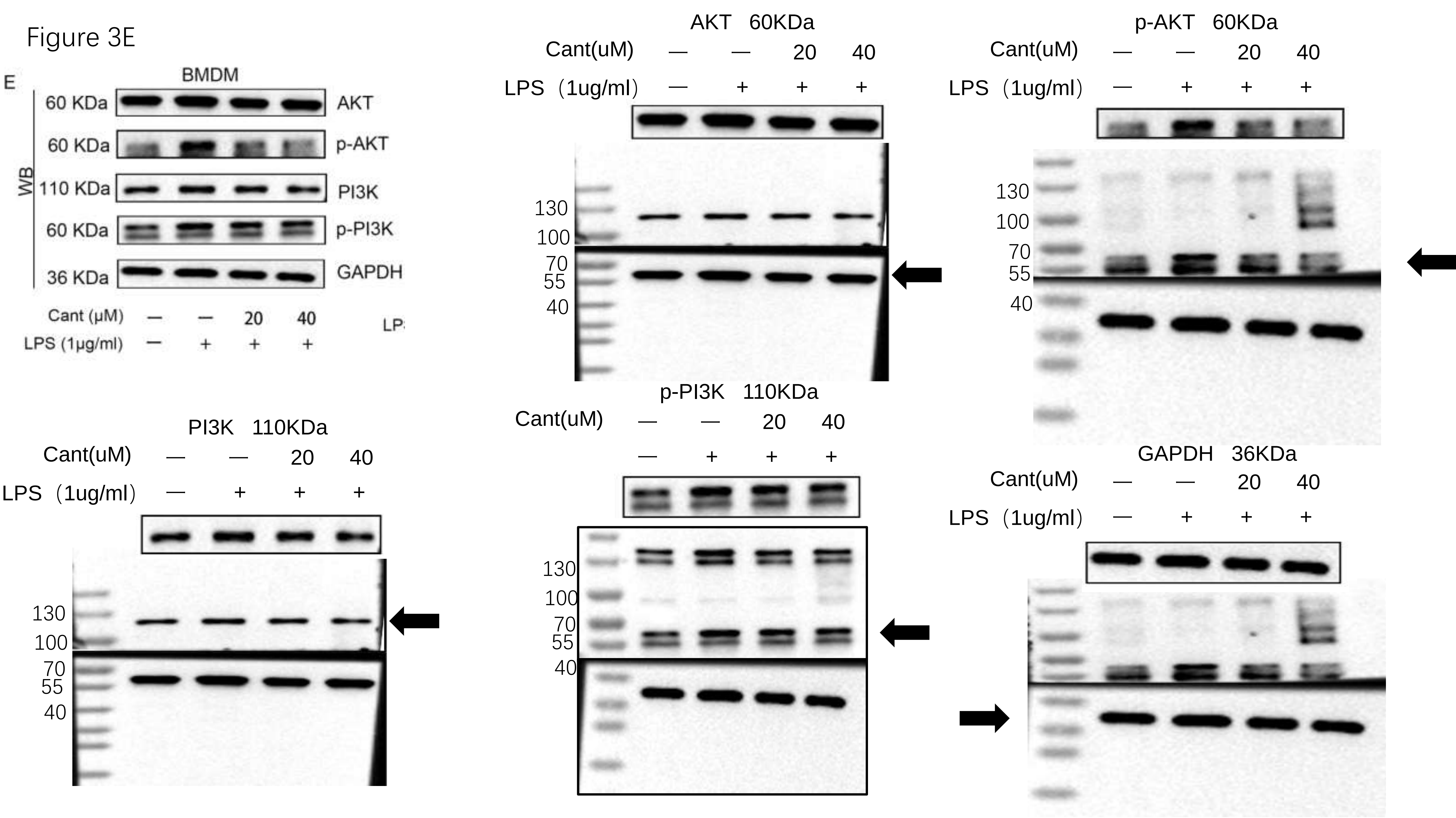

E
BMDM
WB
60 KDa AKT
60 KDa p-AKT
110 KDa PI3K
60 KDa p-PI3K
36 KDa GAPDH
Cant (μM) — — 20 40
LPS (1μg/ml) — + + +
AKT 60KDa
Cant(uM) — — 20 40
LPS (1ug/ml) — + + +
130
100
70
55
40
p-AKT 60KDa
Cant(uM) — — 20 40
LPS (1ug/ml) — + + +
PI3K 110KDa
Cant(uM) — — 20 40
LPS (1ug/ml) — + + +
p-PI3K 110KDa
Cant(uM) — — 20 40
— + + +
GAPDH 36KDa
Cant(uM) — — 20 40
LPS (1ug/ml) — + + +

Figure 4A

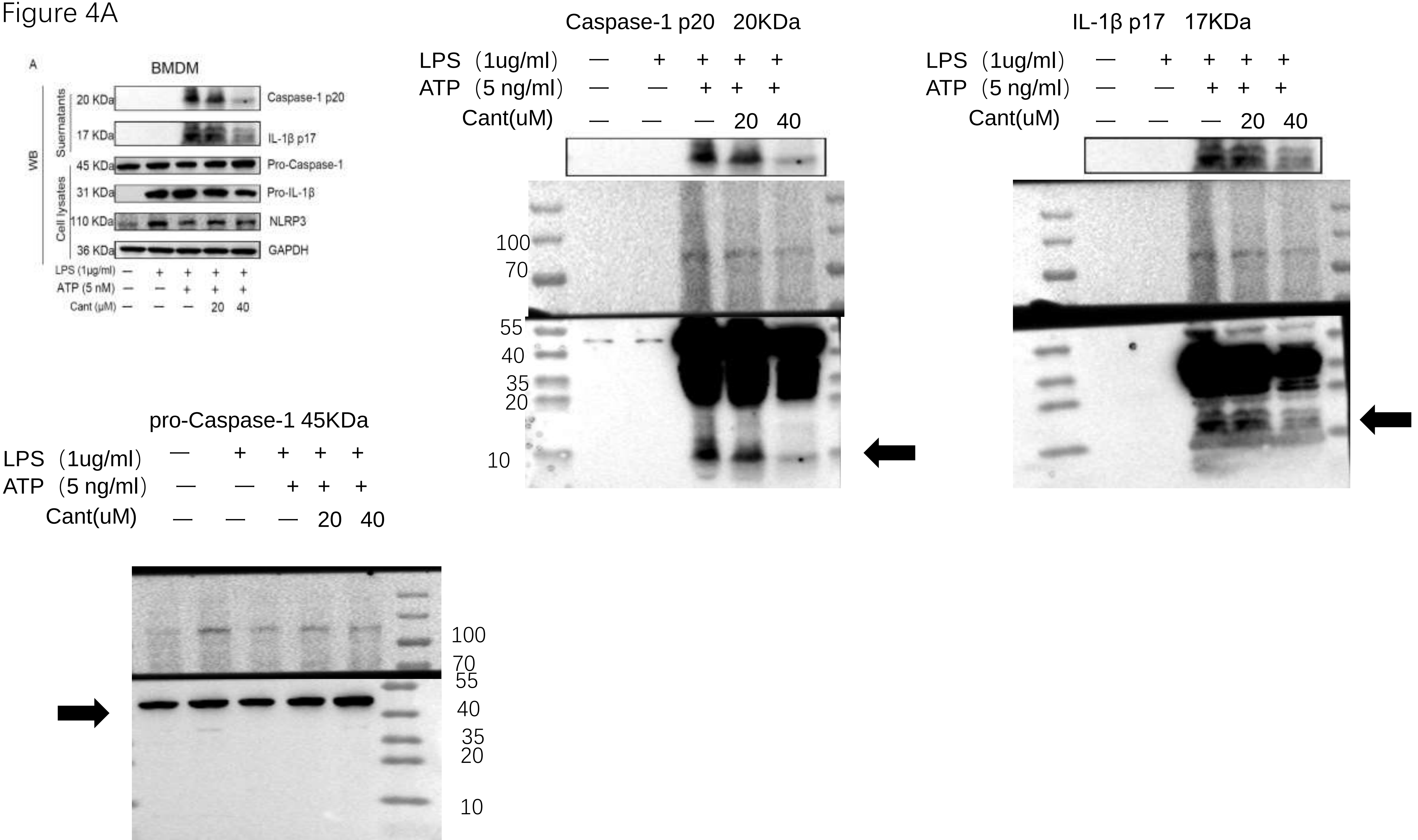
A
BMDM
WB
Suernatants
Cell lysates
20 KDa
17 KDa
45 KDa
31 KDa
110 KDa
36 KDa
Caspase-1 p20
IL-1β p17
Pro-Caspase-1
Pro-IL-1β
NLRP3
GAPDH
LPS (1μg/ml) — + + + +
ATP (5 nM) — — + + +
Cant (μM) — — — 20 40
Caspase-1 p20 20KDa
LPS (1ug/ml) — + + + +
ATP (5 ng/ml) — — + + +
Cant(uM) — — — 20 40
100
70
55
40
35
20
10
IL-1β p17 17KDa
LPS (1ug/ml) — + + + +
ATP (5 ng/ml) — — + + +
Cant(uM) — — — 20 40
pro-Caspase-1 45KDa
LPS (1ug/ml) — + + + +
ATP (5 ng/ml) — — + + +
Cant(uM) — — — 20 40
100
70
55
40
35
20
10

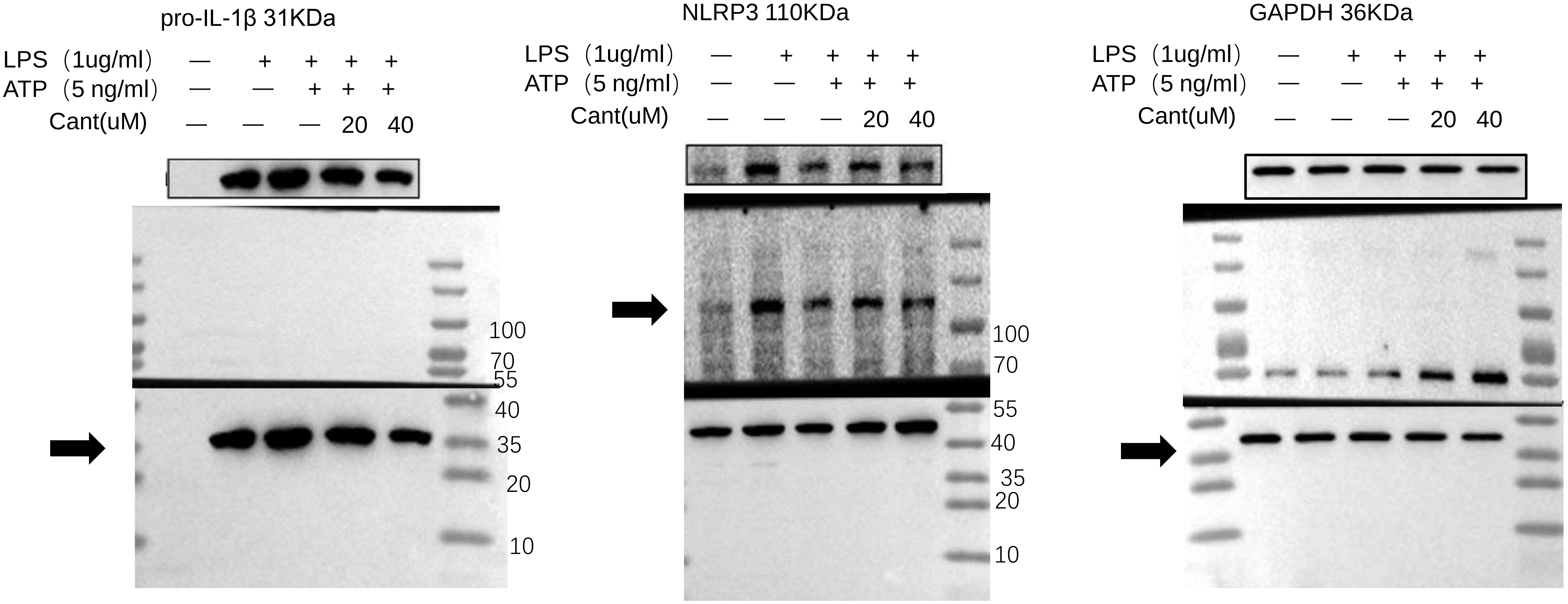

pro-IL-1β 31KDa
LPS（1ug/ml） — + + + +
ATP（5 ng/ml） — — + + +
Cant(uM) — — — 20 40
100
70
55
40
35
20
10
NLRP3 110KDa
LPS（1ug/ml） — + + + +
ATP（5 ng/ml） — — + + +
Cant(uM) — — — 20 40
100
70
55
40
35
20
10
GAPDH 36KDa
LPS（1ug/ml） — + + + +
ATP（5 ng/ml） — — + + +
Cant(uM) — — — 20 40

Figure 4C

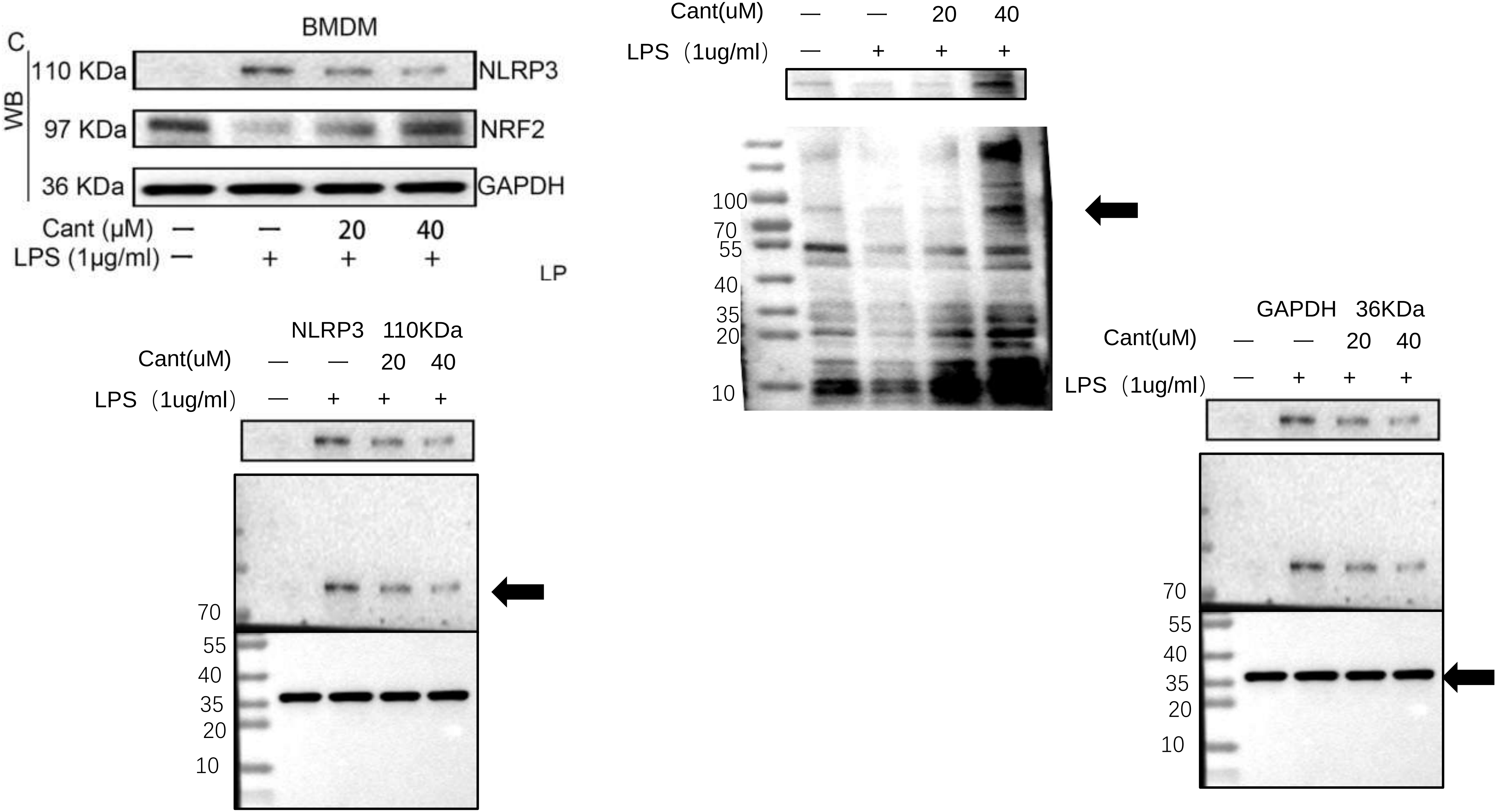
BMDM
C
WB
110 KDa
NLRP3
97 KDa
NRF2
36 KDa
GAPDH
Cant (μM) — — 20 40
LPS (1μg/ml) — + + +
LP
NLRP3 110KDa
Cant(uM) — — 20 40
LPS（1ug/ml） — + + +
70
55
40
35
20
10
NRF2 96KDa
Cant(uM) — — 20 40
LPS（1ug/ml） — + + +
100
70
55
40
35
20
10
GAPDH 36KDa
Cant(uM) — — 20 40
LPS（1ug/ml） — + + +
70
55
40
35
20
10
